# Different Mutations Endowing Resistance to Acetyl-CoA Carboxylase Inhibitors Results in Changes in Ecological Fitness of *Lolium rigidum* Populations

**DOI:** 10.3389/fpls.2017.01078

**Published:** 2017-06-22

**Authors:** Maor Matzrafi, Ofri Gerson, Baruch Rubin, Zvi Peleg

**Affiliations:** The Robert H. Smith Institute of Plant Sciences and Genetics in Agriculture, The Robert H. Smith Faculty of Agriculture, Food and Environment, The Hebrew University of JerusalemRehovot, Israel

**Keywords:** ACCase inhibitors, competition, germination, *Lolium rigidum*, ecological fitness penalty, target-site resistance

## Abstract

Various mutations altering the herbicide target site (TS), can lead to structural modifications that decrease binding efficiency and results in herbicide resistant weed. In most cases, such a mutation will be associated with ecological fitness penalty under herbicide free environmental conditions. Here we describe the effect of various mutations, endowing resistance to acetyl-CoA carboxylase (ACCase) inhibitors, on the ecological fitness penalty of *Lolium rigidum* populations. The TS resistant populations, MH (substitution of isoleucine 1781 to leucine) and NO (cysteine 2088 to arginine), were examined and compared to a sensitive population (AL). Grain weight (GW) characterization of individual plants from both MH and NO populations, showed that resistant individuals had significantly lower GW compared with sensitive ones. Under high temperatures, both TS resistant populations exhibited lower germination rate as compared with the sensitive (AL) population. Likewise, early vigor of plants from both TS resistant populations was significantly lower than the one measured in plants of the sensitive population. Under crop-weed intra-species competition, we found an opposite trend in the response of plants from different populations. Relatively to inter-population competition conditions, plants of MH population were less affected and presented higher reproduction abilities compared to plants from both AL and NO populations. On the basis of our results, a non-chemical approach can be taken to favor the sensitive individuals, eventually leading to a decline in resistant individuals in the population.

## Introduction

Major parts of cultivated land in the world (∼70%) are occupied by cereal crop-plants such as bread wheat (*Triticum aestivum*), corn (*Zea maize*), and rice (*Oryza sativa*) (FAOSTAT, 2016). Rigid ryegrass (*Lolium rigidum* Gaud.) is among the worldwide most noxious grass weed species infesting winter cereal-crops worldwide (Heap, 2017). For example, field studies in Australia have shown that *L. rigidum* can cause yield reductions of more than 40% (Pannell et al., 2004). Chemical control is the most cost efficient method to reduce the yield losses associated with weeds infestation. However, in recent years, increasing abundance of herbicide-resistant weed populations endanger food security for the ever-increasing world population. Herbicide resistance of *Lolium* species has been reported in various habitats; agricultural fields, orchards, vineyards, road sides, and more (Heap, 2017). Notably, *L. rigidum* was found to be the species that developed resistant to the highest number of different modes of action (MOA; Heap, 2017), which can be consequence of the obligate outcrossing nature of this specie (Mccraw et al., 1983).

Fatty acid biosynthesis is a crucial stage in the formation of different organelles, waxes and other secondary metabolites (Harwood, 1988). Acetyl CoA carboxylase (ACCase) is a key enzyme in the first step of fatty acids synthesis, it catalysis the formation of malonyl-CoA from acetyl-CoA (Roesler et al., 1997). Two isoforms of ACCase, heteromeric and homomeric, are present in plants. While most plants have both forms in different cell compartments (cytosol and plastids), in the Gramineae species, only the homomeric form of the enzyme is present in both compartments (Sasaki and Nagano, 2004). This fact facilitated the creation of this unique group of selective and highly efficient ACCase inhibitors targeted to damage only grass weeds. Three different chemical groups are classified as ACCase inhibitors: aryloxyphenoxypropionates (AOPP), cyclohexanediones (CHD), and phenylpyrazoline (PPZ) (HRAC, 2017). Their mechanism of action, blocking only the *in vivo* activity of the plastidic homomeric ACCase form, exist in grass weeds only (Sasaki and Nagano, 2004).

Herbicide resistance has two sub categories of target site (TS) and non-target site (NTS) resistance. TS resistance is the result of structural modification, or over-expression of a specific gene encoding for the target protein (Délye et al., 2013a). NTS resistance can occur due to reduced translocation (González-Torralva et al., 2012), modification in subcellular distribution (Kleinman and Rubin, 2016), herbicide detoxification (Matzrafi et al., 2016) or other mechanisms (reviewed by Délye et al., 2013a). To date, seven different substitution were reported in the ACCase gene sequence, which result in various structural modifications reducing the binding efficiency of the herbicide molecule reviewed by Kaundun, 2014).

Fitness penalty associated with herbicide resistance was previously demonstrated in various weed species for both TS (Menchari et al., 2008; Shergill et al., 2016; Frenkel et al., 2017) and NTS (Vila-Aiub et al., 2005a,b) resistance mechanisms. In most cases, TS resistance to ACCase inhibitors via alteration of the ACCase enzyme will inevitably lead to a penalty in plant performance. Changes in morpho-physiological traits such as seed germination rate (Vila-Aiub et al., 2005b), biomass production (Menchari et al., 2008) and reproductive abilities (Papapanagiotou et al., 2015), were reported in association with TS resistance to ACCase inhibitors. The level of fitness penalty can be affected by different mutations and plant species. A substitution of isoleucine 2041 to asparagine in the *Hordeum glaucum* ACCase gene, resulted in reduced vegetative biomass and seed production, while substitution of isoleucine 1781 to leucine/valine did not carry any fitness penalty (Shergill et al., 2016). On the other hand, in *Setaria viridis* alteration in position 1781 led to higher fitness in the resistant (i.e., mutant) plant compared to the wild type (Wang et al., 2010).

While the level of herbicide resistant associated with TS mutations is not affected by environmental conditions (Vila-Aiub et al., 2009), we hypothesize that environmental conditions will have a major effect on the developmental and reproductive performances of TS resistance plants, under herbicide-free conditions. Here we characterized the differences in ecological fitness penalty caused by TS mutations (isoleucine 1781 to leucine and cysteine 2088 to arginine) in *L. rigidum* populations. Our specific aims were to: (**i**) define the effect of different mutations in the ACCase gene sequence on grain features, (**ii**) test the effect of environmental conditions on ecological fitness penalty, and (**iii**) characterize the competition ability of each population.

## Materials and Methods

### Plant Material

Seeds of the sensitive (Alumim, AL) *L. rigidum* population were collected from an organic wheat field where no herbicides were applied. Seeds of two TS resistant *L. rigidum* populations, Ma’oz Haim (MH, substitution of isoleucine 1781 to leucine) and Nahal Oz (NO, substitution of cysteine 2088 to arginine), were collected follow failures of ACCase inhibitors to control *L. rigidum* plants: clodinafop-propargyl in wheat field and clethodim in a carrot field, respectively. In each field, seeds from 20 to 30 random mature plants were collected into a paper bag and termed as “population”. The seeds were separated, air-dried and stored at 4°C until used.

Seeds from each population were germinated in trays filled with a commercial growth mixture (Tuff Merom Golan, Israel). Trays were placed in a controlled growth room (16°C) to break the seeds’ dormancy, and at two-leaf stage, seedling were transplanted into plastic pots (7 cm × 7 cm × 6 cm, one plant per pot) containing the same growth mixture. Plants were placed in a controlled greenhouse (18/25°C night / day) and watered as needed. TS-resistant plants from MH and NO populations were selected three times with the same herbicide that was used in the field (clodinafop-propargyl and clethodim, respectively). Individual plants that survived herbicide application (5–6) from each population were grown in cages covered with air breathing nylon screens to prevent foreign pollination. Plants of the sensitive population (AL) were grown under the same conditions without herbicide application. Seeds of all three populations were harvested, air-dried and stored at 4°C as described above. The same germination procedure was used for all experiments.

### Response of *L. rigidum* Populations to ACCase Inhibitors

Three herbicides, representing the three chemical groups of the ACCase inhibitors were used for this study, when X = the recommended rate. Aryloxyphenoxypropionate (Fop) – diclofop-methyl (Iloxan^®^, 360 g L^-1^ EC, Bayer, Germany; X = 720 g a.i. ha^-1^), phenylpyrazole (Den) – pinoxaden (Axial^®^, 45 g L^-1^ + cloquinotocet-mexyl 11.25 g L^-1^ EC, Syngenta, Switzerland; X = 30 g a.i. ha^-1^) and cyclohexandione (Dim) – cycloxydim (Focus^®^, 100 g L^-1^ EC, BASF; Germany, X = 100 g a.i. ha^-1^), were used. Plants (3–4 leaves stage) sprayed with increasing rates (0, 0.25X, 0.5X, X, 2X, 4X, and 8X) of the three herbicides, in order to quantify the level of resistance under controlled conditions. Herbicides applied using a chain-driven sprayer delivering 300 L ha^-1^. Plant shoot fresh weight (FW) was recorded 21 days after treatment (DAT).

### Target Site Resistance Validation

Fresh leaf tissue (∼200 mg) of plants from both MH and NO populations that survived herbicide application, and from sensitive control plants of AL population, were sampled and kept under –80°C until used. DNA extracted using the Puregene DNA isolation kit (Gentra, MN, United States) according to the manufacturer’s instructions. For sequence analysis, primers were designed using known sequence of *L. rigidum* (DQ184640.1), as described previously (Matzrafi et al., 2014 and Supplementary Table S1). Specific regions in the ACCase gene sequence were amplified and PCR products were sequenced to locate the common point mutations that might endow TS resistance. Sequence analyses and alignment were performed using Bioedit software (Hall, 1999). The obtained sequences were compared to the known ACCase gene sequence of *Alopecurus myosuroides* (AJ3107671).

### Grain Characterization

Sixty grains from TS resistant (MH and NO) and sensitive (AL) *L. rigidum* populations were weighed on a microbalance (M2P, Sartorius, Göttingen, Germany) to obtain grain weight (GW). The same 60 grains from each population were photographed using a binocular (SZX16, Olympus, Tokyo, Japan) and analyzed to obtain grain area parameters. Image analysis and processing were performed using the Matlab software (MathWorks, Natick, Massachusetts, United States) and the public-domain software ImageJ^1^ (NIH). Pictures were converted into gray scale, and mean gray value (MGV) was calculated from the average gray scale value of pixels in the selected area using equation 1:

MGV=0.2989×R+0.5870×G+0.114×B

where R, G, and B stands for the three spectral regions: red, green, and blue, respectively. Based on the MGV, a threshold was selected to include all grain pixels (MGV > 0.13), and minimum size of 5000 pixels was defined to create non-disturbed analysis. Suitable pixels gained the value of 1 and transformed into a binary picture as white pixels. The ratio between the lengths of each pixel/micron was calculated using ImageJ software so that the data on white pixels in the picture were converted into grain area according to equation 2:

$$16\mu m^{2}=0.25{pixel}_{\left(length\right)}/1\mu m\to {pixel}_{area}$$

Follow the grains weight analysis, all 180 seeds (60 from each population) were sown, one per pot (7 cm × 7 cm × 6 cm), and placed in a greenhouse (19/25°C, night/day). At 3–4 leaves stage, plants were sprayed with diclofop-methyl (2X). Survival rate (plants that produced new leaves) was recorded 21 DAT.

### Characterization of Seed Emergence Rate

Five seeds from each population were placed into one pot (9 cm × 9 cm × 10 cm, filled with growth mixture) and covered with a thin layer of the same mixture (0.25 cm). Pots were placed in a controlled dark growth chamber under two temperature regimes: control conditions (10/16°C night/day) and high temperature (19/25°C). Three replicates were used for each population × temperature combination. Seedling emergence (appearance of the coleoptile) recorded daily over 14 days.

### Characterization of Inter-Population and Intra-species Competition

Seedlings of the sensitive (AL) and resistant (MH and NO) *L. rigidum* populations, and bread wheat *cv*. Zahir (Hazera, Israel) were transplanted into 4.3 L pot (25cm × 15cm × 11.5cm) filled with a mixture of 80% brown–red degrading sandy soil (Rhodoxeralf; composed of 76% sand, 8% silt and 16% clay) and 20% of growth mixture. Each population was arranged either alone (i.e., inter-population competition) or together with wheat (i.e., intra-species competition), with total 28 plants per pot (to mimic field density of 746 plants/m^2^), three replications for each combination. Plants were grown in a controlled greenhouse (16/25°C night/day) under short day (10 h) conditions.

Pots were photographed with an RGB digital camera (Canon PowerShot SX20 IS, Canon, Melville, NY, United States), in an overhead view (53 cm from the camera lens to the surface of the box). Plants were photographed over 35 days in intervals of 7 days. Images were analyzed to estimate vigor differences between different populations using the Matlab software. In any 8-bit JPEG image setting of different color components of individual pixel was done using the values obtained from each of the three color channels; red (R), green (G), and blue (B). To quantify the vegetation area cover, we cropped the image to the pot size and created a binary matrix in the same size. Pixels that had the highest value in the green channel (G > R and G > B) were assigned to the array 1 in the binary matrix, others (G < B or G < R) were assigned as 0. The proportion of the vegetation area in the pot was calculated as sum of 1 array in the matrix divided by the size of the matrix as described in equation 3:

$$\%\text{Foliage cover=}\frac{\text{black pixels in selected area}}{\text{total selected area}}\times 100$$

Plants from both competition categories were harvested 64 days after transplanting, the numbers of tillers and spikes were recorded and all aboveground biomass was oven-dried (80°C for 48 h) and then weighed. Since each population had different plant architecture, in order to be able to compare between populations under the intra-species competition, values were calculated relatively to inter-population competition standards.

### Statistical Analyses

The JMP Pro 13 (SAS Institute Inc., Cary, NC, United States) was used for all statistical analyses. Differences between treatments were examined using different tests as specified in each experiment. Analysis of variance (ANOVA) performed to examine the effect of single variants and the interactions between different treatments. Dose-response curves were constructed by plotting the shoot DW data 21 DAT, from the different populations as a percentage of untreated control (UTC). All the data including, seed emergence, foliage cover, GW, and area, were analyzed using SigmaPlot (ver. 10) software (Systat Software Inc., San Jose, CA, United States). A nonlinear curve model [sigmoidal logistic, three parameters; Seefeldt et al., 1995] was adjusted to analyze the effects of the tested herbicides in the different experiments, as described in Equation 4:

$$\text{Y=}\frac{a}{1+{\left(\frac{x}{x_{0}}\right)}^{b}}$$

In the model, if *b* > 0, then *a* describes the upper limit of Y. X_0_ = ED_50_ and *b* describes the slope of the curve in ED_50_. The resistance index (RI) was calculated as the ratio of the ED_50_ value of the resistant accession to the ED_50_ of the sensitive one.

## Results

### Herbicide Resistance in *Lolium rigidum* Populations

**FIGURE 1:**
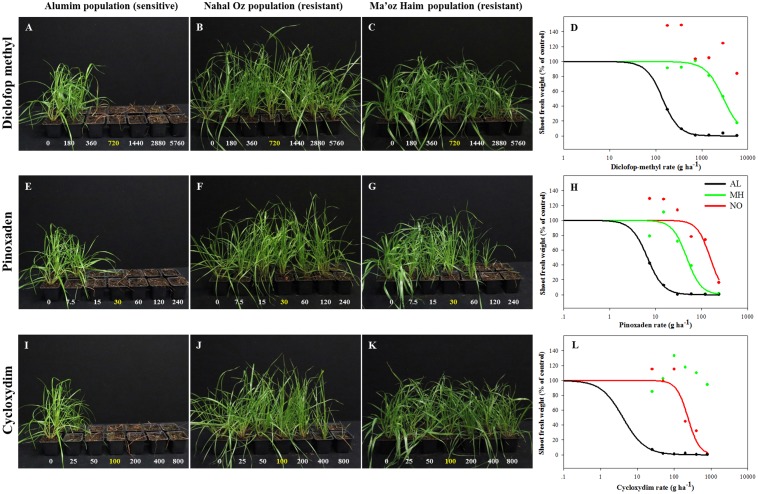
Effect of application of increasing rates of different ACCase inhibitors: diclofop-methyl **(A–D)**, pinoxaden **(E–H)**, and cycloxydim **(I–L)**, on the survival rate and shoot fresh weights of plants from sensitive (AL, black) and target-site resistant (MH, green and NO, red) *Lolium rigidum* populations.

Individuals from all three *L. rigidum* populations were analyzed for their response to herbicides from three chemical classes of ACCase inhibitors: diclofop-methyl, pinoxaden, and cycloxydim. Plants of the sensitive population (AL) did not survive treatments with more than one quarter of the recommended rate of all three herbicides (diclofop methyl = 180, Pinoxaden = 7.5, and cycloxydim = 25 a.i. ha^-1^) (**Figure 1**). Plants from both MH and NO populations showed high survival rates in response to increasing rates of diclofop-methyl up to 8X (**Figures 1B–D**). Additionally, plants from NO population did not show 50% decrease in shoot FW and ED_50_ value could not be extracted under diclofop-methyl treatment (**Figure 1D** and Supplementary Table S2). The same trend (no ED_50_) was shown in the response of plants from MH population to cycloxydim (**Figure 1L** and Supplementary Table S2). Under pinoxaden treatment, plants from MH and NO populations showed high RI values (7.14 and 23.1, respectively; **Figures 1E–H** and Supplementary Table S2).

DNA sequencing of specific sections in the ACCase gene showed substitutions in both heterozygote and homozygote forms in plants from MH and NO populations. MH plants showed substitution of isoleucine 1781 to leucine, and NO plants had substitution of cysteine 2088 to arginine (Supplementary Figure S1).

### Grain Characterization

**FIGURE 2:**
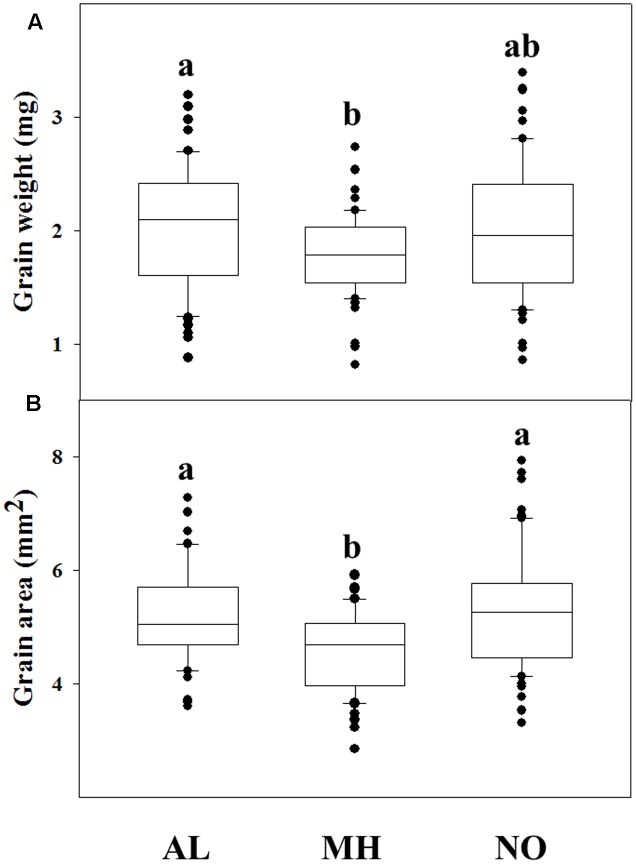
Grain parameters of 60 individuals from different *Lolium rigidum* populations. **(A)** grain weight and **(B)** grain area in of the sensitive (AL) and target-site resistant (MH and NO) *L. rigidum* populations. Different letters indicate significant differences between populations by Tukey-HSD (*P* ≤ 0.05).

A correlation between fitness penalties and grain characteristics have been reported for various plant species (e.g., Wang et al., 2010; Shergill et al., 2016). Comparison of grains from each population revealed high differences among grains of TS populations compared to grains of the sensitive one. GW and surface area of individuals from MH population was significantly lower compared to grains of AL population (1.79 mg and 4.55 mm^2^ *vs.* 2.05 mg and 5.19 mm^2^, respectively; **Figures 2A,B**). The NO population grains showed lower values, but not significant, in measured GW (1.99 mg) and area (5.28 mm^2^), compared to grains of AL population (**Figures 2A,B** and Supplementary Table S3).

**FIGURE 3:**
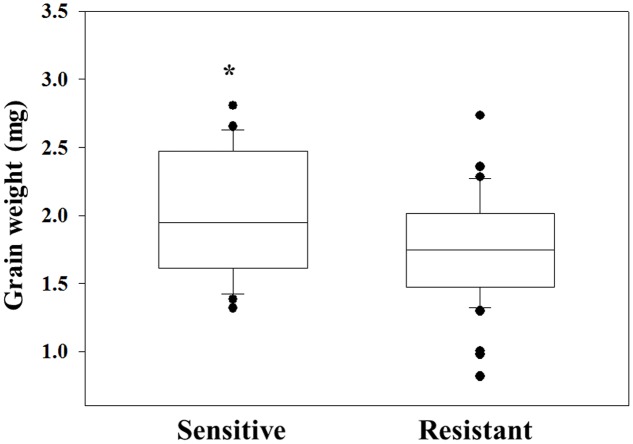
Grain weight of herbicide sensitive (AL) and target-site resistant (MH and NO) *Lolium rigidum* populations. Data is mean [sensitive (*n* = 22) and resistant (*n* = 45)]. ^∗^indicate significant differences between the two groups using Student *t*-test at *P* ≤ 0.05.

The same grains were germinated in pots and sprayed at 3–4 leaf stage with 2X of diclofop-methyl. Twenty-one DAT plant survival rate and FW were recorded. Grain of plants that were found to be resistant to diclofop-methyl showed significantly lower GW than what was measured in sensitive plants (1.75 mg *vs.* 2 mg, respectively, **Figure 3** and Supplementary Table S2). Notably, only 38.09% of the individuals in the NO population were diclofop-methyl resistant as compared to 94.74% in the MH population (Supplementary Table S4).

### Seedling Emergence Rate

**FIGURE 4:**
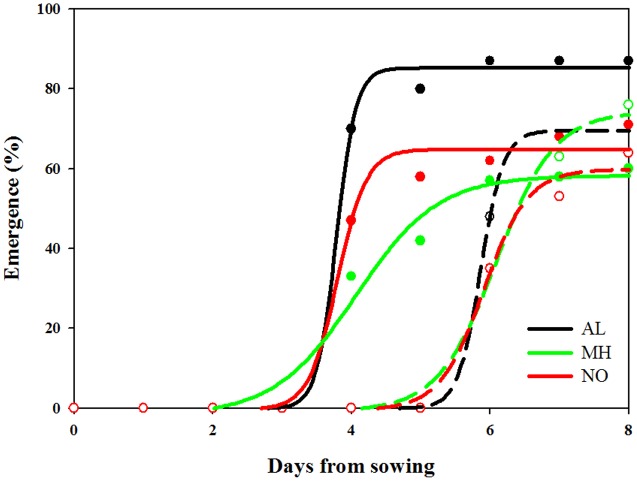
Effect of temperature on seedling emergence rate of sensitive (AL, black) and target-site resistant (MH, green and NO, red) *Lolium rigidum* populations. Temperature regimes: control 10/16°C (open circles, dash line) and high temperature treatment 19/25°C (close circles, full line).

In general, seedling emergence in all *L. rigidum* populations started earlier under high (19/25°C) compared to control (10/16°C) temperature regimes (**Figure 4** and Supplementary Table S5). Under high temperatures, seedlings from both AL, and NO populations emerged faster and at higher percentage as compared to seedlings from MH population. Emergence of seeds from all three populations under the favorable temperature regime (control) was similar (**Figure 4** and Supplementary Table S5).

### Effect of Inter- and Intra- Species Competition of Ecological Fitness Penalty

**FIGURE 5:**
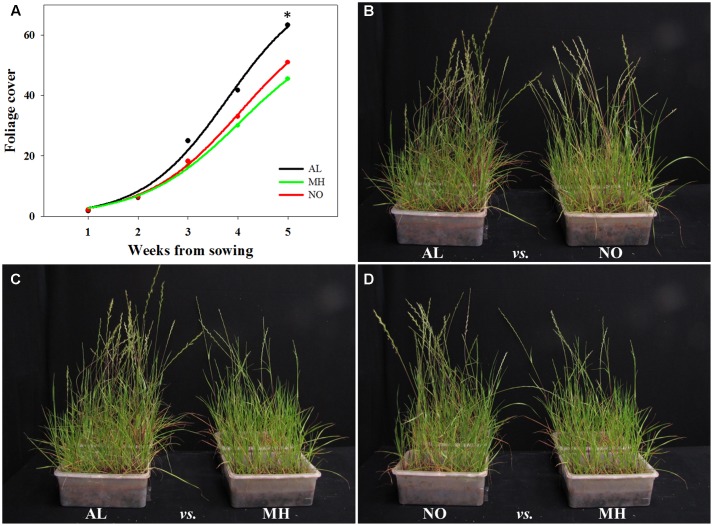
Visual assessment of inter-population competition between sensitive (AL) and target-site resistant (MH and NO) *Lolium rigidum* populations, in an herbicide-free environment. **(A)** Biomass production of sensitive (AL, black) and target-site resistant (MH, green and NO, red) *L. rigidum* populations, estimated every week (total five weeks) by image analysis and presented as foliage cover. Photographs showing the inter-population competition between *L. rigidum* populations: **(B)** AL *vs.* NO, **(C)** AL *vs.* MH and **(D)** NO *vs.* MH. ^∗^indicate significant difference between populations at *P* ≤ 0.05.

TS resistance to various herbicide MOAs had been reported to be associated with reduction in competition abilities, which can affect plants ecological fitness (e.g., Chun et al., 2013; Shergill et al., 2016). Here, plants from TS-resistant and -sensitive populations of *L. rigidum* were tested for their inter-population and intra-species (with wheat) competition abilities. Plants of AL population grown under inter-population competition showed higher foliage cover from the second week of the experiment, eventually leading to significant differences in coverage comparing to plants of both MH and NO populations (63.26 cm^2^ plant^-1^ *vs.* 45.46 cm^2^ plant^-1^ and 50.99 cm^2^ plant^-1^, respectively; **Figure 5A** and Supplementary Table S6).

Under inter-population competition, plants from MH population showed reduced tillers numbers (2.26 *vs.* 3.30 and 3.05 tillers plant^-1^ for AL and NO plants, respectively) and vegetative aboveground growth (10.33 g plant^-1^ *vs.* 16.42 g plant^-1^ and 11.92 g plant^-1^ for AL and NO plants, respectively; **Table 1** and **Figures 5B–D**). Plants from MH population also presented significantly lower productivity with less spikes (0.6 plant^-1^) compared to plants from AL (1.6 plant^-1^) and NO (1.5 plant^-1^) populations (**Table 1**). Furthermore, plants of MH population were shorter (34.9 cm) compared to individuals from both AL and NO (44.0 and 41.5 cm, respectively). Plants from the sensitive population of AL ranked the highest score in each of the morpho-physiological parameters under inter-population competition (**Table 1**).

**Table 1:** The effect of inter-population and intra-species competition, on dry weight biomass production, plant height, number of tillers, and number of spikes, on untreated plants of sensitive (AL) and target-site (TS) resistant (MH and NO) *Lolium rigidum* populations.

Population	Dry weight, g plant^-1^	Plant height, cm	No. of tillers, plant^-1^	No. of spikes, plant^-1^
**Inter-population**				
AL	16.4 ± 1.9a	44.0 ± 3.7a	3.3 ± 0.1a	1.6 ± 0.3a
NO	11.9 ± 2.2a	41.4 ± 3.8a	3.0 ± 0.2a	1.5 ± 0.2a
MH	10.3 ± 1.1a	34.9 ± 3.4a	2.3 ± 0.1b	0.6 ± 0.1b
**Intra-species (relative value^1^)**				
AL	0.06 ± 0.1a	1.0 ± 0.1a	0.9 ± 0.1a	0.8 ± 0.1b
NO	0.11 ± 0.2a	1.1 ± 0.1a	0.8 ± 0.1a	1.0 ± 0.2b
MH	0.05 ± 0.1a	1.2 ± 0.1a	1.0 ± 0.1a	1.6 ± 0.09a


*Different letters indicate significant differences (*P* ≤ 0.05) between plants from three *Lolium rigidum* populations by Tukey LSD test.^1^Intra-species competition calculated as a relative value in comparison to the average value that was recorded for different parameters in the inter-population competition.*

Under intra-species competition of *L. rigidum* and wheat plants, the values of different parameters were calculated as a ratio relative to the inter-population competition. Plants of MH population showed less sensitivity to intra-species competition than plants of AL and NO populations in most parameters (**Table 1**). This advantage was strongly pronounced in differences found in the number of spikes, were plants of MH population showed a significant increase (1.57) in productivity compared to plants from both AL and No population (0.79 and 0.98, respectively; **Table 1**).

## Discussion

ACCase inhibitors have been used in modern agriculture since the 1970s of the previous century and played important role in selective control of grass weeds (Kaundun, 2014). Selection pressure caused by repeated- and/or mis-application unravel different TS and NTS resistance ecotypes (Délye et al., 2013a), and can eventually lead to the evolution of resistant individuals in a field population (Rubin, 1991). Moreover, obligate outcrossing grass weed species, such as *A. myosuroides* and *Lolium* species, contributes to the distribution of these resistance traits (Mccraw et al., 1983; Yang et al., 2008). TS resistance to ACCase inhibitors has been reported to be associated with fitness penalty (Vila-Aiub et al., 2005b; Menchari et al., 2008; Kukorelli et al., 2013). Here we used a comprehensive approach to characterize the effect of different mutations in the ACCase gene on the ecological fitness of resistant *Lolium* species populations throughout the plants’ life cycle.

Examining of grains from *Lolium* populations with different herbicide response, individuals of the sensitive AL population showed the highest GW compared to ones from both MH and NO populations (**Figure 2**). Moreover, the same trend of lower GW was found among sensitive and resistant plants from the same TS resistant population. Notably, Sabet Zangeneh et al. (2016) reported that TS resistance *L. rigidum* populations (Ile 1781 to Leu and Ile 2041 to Asn) had no significant differences in thousand seeds’ weight compared with a sensitive population. Seed weight can affect the level of dormancy, germination rate and early seedling establishment. Under agro-ecosystems, species with larger seed weight will have advantage coping with adverse establishment conditions such as burial, competition, low soil moisture, and nutrients imbalance (e.g., Leishman, 2000; Van Etten et al., 2016). In agreement, the sensitive population (AL), with greater seed weight showed faster seed emergence under both temperature regimes compared to both TS resistant populations (Supplementary Table S5). Smaller grains size of resistant plants can also explain the decrease in their inter-population competitions abilities (**Figure 3**), as was also reported previously (Van Etten et al., 2016).

Analysis of the association between seed weight and herbicide response in the two TS resistant populations showed that while most (94.7%) of plants from MH population were resistant, only 38.1% of plant from the NO population were recorded as such (Supplementary Table S4). Interestingly, all individuals of NO population that were treated with diclofop-methyl in the dose response experiments had survived the treatment. Furthermore, compared with the MH population, higher resistance was found in NO population at the maximum rate of 8X (**Figures 1B–D** and Supplementary Table S2). These results suggest that the NO population is in transition phase compared to MH population that is more uniform in its herbicide response at the population level.

Weed competitiveness is associated with resource capture, for example light interception that is considered major limiting factor for weed growth under conventional cropping systems (Holt, 1995). Environmental factors such as: radiation, temperature and competition were previously shown to affect seed dormancy (Sbatella and Wilson, 2010), grain yield production (Frenkel et al., 2017) and fertility (Gramshaw, 1972; Pedersen et al., 2007). Plants’ canopy height has been suggested as good indicator of light interception, especially for grasses (Cudney et al., 1991; Seavers and Wright, 1999). Plants from MH and NO populations showed reduce plant height, lower biomass production and reproductively compared to plants of AL population (**Table 1**). This trend is further emphasized by the observed accelerated vegetative development and number of tillers/spikes of AL plants compared to plant of TS-resistant populations (**Figure 5C** and **Table 1**).

The sensitive population (AL) exhibited higher abilities compared to the TS resistant MH population, in term of seedling vigor, plant height, biomass and reproductive capacities (**Table 1**). Interestingly, these advantages were reduced under changing competition and temperature conditions, suggesting high phenotypic plasticity as consequence of genotype × environment interactions. The TS resistant population of MH showed less phenotypic plasticity under changing environmental conditions. It can be proposed that narrow genetic variation, caused by 94% resistant individuals, enables less plasticity in the response to environmental changes. Under competition with wheat plants, reproductive abilities of MH plants were less affected compared to both AL and NO plants (**Table 1**). This specific population has been subjected to a strong selection for ACCase inhibitors alongside competition with wheat plants. It may have facilitated the evolution of a dual advantage, herbicide resistance/crop-weed competition abilities. Plants competition play a key role in weed community, mainly due to limited resources (Navas, 2012). The ability of a plant to grow and survive in response to resource depletion due to competition with neighboring plants is pronounced by its competitive abilities.

Normally, ACCase inhibitors are applied in the beginning of the growing season at a critical period for weed control (Knezevic et al., 2002). Our results suggests, that the sensitive plants that emerge earlier under high temperatures are exposed and controlled by the herbicide, whereas resistant individuals will germinate later in season, and thus, will eventually escape herbicide application. This can be further exacerbated under the projected climate change scenarios, which predict an aggravation in the intensity and frequency of extreme events, such as temperature fluctuations (Easterling et al., 2000). Environmental factors such as temperature and water availability are highly important factors in seed germination, both rate and percentage (Alvarado and Bradford, 2002). Membrane composition was found to play a key role in the response of seeds to environmental cues which influence the germination processes (Nishida and Murata, 1996). The activity of the ACCase enzyme play a crucial role in fatty acid biosynthesis thus on lipid content, membrane structure, and other essential cell components (Harwood, 1988; Price et al., 2003). It can be suggested that the differences found between sensitive and TS-resistant population in seedling ability to emerge is associated with changes in fatty acids composition derived by the altered ACCase enzyme activity. In previous studies, high lipid content was shown to be correlated with higher and faster germination rate in several weed species (Gardarin et al., 2011). It was also suggested that seeds with higher soluble content, such as higher lipid content, can have altered water adsorbent ability than other seeds and will eventually germinate faster (Nonogai, 2006). More studies are needed to test the interaction between the ACCase alteration, fatty acid composition and plant ecological fitness.

## Conclusion

Fitness penalty is inevitable phenomenon associated with TS modifications (Vila-Aiub et al., 2009). Moreover, our results, as well as previous studies (Menchari et al., 2008; Délye et al., 2013b; Jang et al., 2013), demonstrate that different substitutions in the same target gene would not necessarily have the same effect on the level of fitness penalty. In the current study, we show that while under specific environmental conditions the effect of mutation in the target gene on plant fitness can be minimal, under altered environmental conditions the level of fitness penalty can increased. Thus, we suggest that in order to understand the effect of TS resistance on fitness of weed population, plants need to be tested under different environmental conditions and growth stages.

Understanding the interaction between different TS-resistance ecotypes and environmental conditions could serve as a powerful tool for developing improved weed management techniques. Using integrated weed management combining non-chemical and chemical approached that can favor the sensitive ecotypes, a gradual dilution of the resistance individual proportion in the weed population can be achieved.

## Author Contributions

MM, OG, and ZP designed the experiments. MM and OG conducted the experiment. MM, OG, BR, and ZP analyzed data and wrote the paper. All authors read and approved the manuscript.

## Conflict of Interest Statement

The authors declare that the research was conducted in the absence of any commercial or financial relationships that could be construed as a potential conflict of interest. The reviewer JN and handling Editor declared their shared affiliation, and the handling Editor states that the process met the standards of a fair and objective review.

## Footnotes

1 http://rsb.info.nih.gov/ij
