# Supplementary material for: Different Mutations Endowing Resistance to Acetyl-CoA Carboxylase Inhibitors Results in Changes in Ecological Fitness of Lolium rigidum Populations
Source: Front Plant Sci. 2017 Jun 22;8:1078. doi: 10.3389/fpls.2017.01078 (PMC5479926; doi:10.3389/fpls.2017.01078)
Supplement: Supplementary file 2 [file Presentation_1.PDF]

Supplementary Material

Different Mutations Endowing Resistance to Acetyl-CoA Carboxylase Inhibitors Results in a Unique Shift in Ecological Fitness of Lolium rigidum Populations

Maor Matzrafi, Ofri Gerson, Baruch Rubin and Zvi Peleg

A

```
                                  1770      1780      1790
                               .|....|....|....|....|....|.
Alopecurus myosuroides         WVIDSVVGKEDGLGVENIHGSAAIASAY
Lolium rigidum (AL)            ............................
Lolium rigidum (NO)            ............................
Lolium rigidum (MH)            .................L..........
Lolium rigidum (MH)            .................X..........
```

B

```
                              2070      2080      2090
                               |....|....|....|....|....|..
Alopecurus myosuroides         RGGAWVVIDSKINPDRIECYAERTAKGN
Lolium rigidum (AL)            ............................
Lolium rigidum (NO)            ..................R.........
Lolium rigidum (NO)            ..................X.........
Lolium rigidum (MH)            ............................
```

Figure S1. Alignment of partial sequences of the *ACCase* gene in *Lolium rigidum* populations. Partial sequences including both 1781 (A) and 2088 (B) substitutions found in plants of TS resistant *L. rigidum* populations.
